# Taking the bait: Developing a bait delivery system to target free‐ranging crocodiles and varanid lizards with a novel conservation strategy

**DOI:** 10.1002/ece3.8933

**Published:** 2022-06-02

**Authors:** Abhilasha Aiyer, Bunuba Rangers, Tina Bell, Richard Shine, Ruchira Somaweera, Miles Bruny, Georgia Ward‐Fear

**Affiliations:** ^1^ School of Life and Environmental Sciences University of Sydney Sydney New South Wales Australia; ^2^ Bunuba Dawangarri Aboriginal Corporation Fitzroy Crossing Western Australia Australia; ^3^ 7788 School of Natural Sciences Macquarie University Sydney New South Wales Australia; ^4^ Stantec Australia Perth Western Australia Australia; ^5^ School of Biological Sciences University of Western Australia Crawley Western Australia Australia; ^6^ Department of Biodiversity, Conservation and Attractions Wanneroo Western Australia Australia

**Keywords:** *Bufo marinus*, conditioned taste aversion, *Crocodylus*, invasive species, *Varanus*

## Abstract

In tropical Australia, conditioned taste aversion (CTA) can buffer vulnerable native predators from the invasion of a toxic prey species (cane toads, *Rhinella marina*). Thus, we need to develop methods to deploy aversion‐inducing baits in the field, in ways that maximize uptake by vulnerable species (but not other taxa). We constructed and field‐tested baiting devices, in situ with wild animals. Apparatus were set next to waterbodies and baited concurrently at multiple locations (over water, water's edge, and on the bank). Baits were checked and replaced twice daily during the trial; remote cameras recorded visitation by native predators. Bait longevity was compared at sun‐exposed and shaded locations over 12 h. The strength required to remove baits from apparatus was measured in varanids and crocodiles. The device promoted high rates of bait uptake by freshwater crocodiles (47% baits consumed), varanid lizards (19% baits consumed), and non‐target taxa (34% baits consumed). Targeting specific predators can be achieved by manipulating bait location and time of deployment, as well as the force required to dislodge the bait. Crocodiles were best targeted with over‐water baits, whereas varanid lizards preferred baits located at the edges of waterbodies. When testing bait longevity in ambient conditions, during the daytime baits desiccated fully within 12 h, and faster in the sun than in the shade. Based on studies using captive animals, the “pulling force” strength of reptilian predators scaled with body size and was greater in crocodiles than in varanid lizards. We present the first conservation baiting protocol designed specifically for reptiles. Our results demonstrate the feasibility of widespread and taxon‐specific deployment of aversion‐inducing baits to buffer the impacts of invasive cane toads, and our methods are applicable (with modification) to other research and management programs globally.

## Graphical abstract

Conditioned taste aversion can buffer vulnerable native predators from the invasion of a toxic prey species (cane toads, *Rhinella marina*) and we need ways to deploy aversion‐inducing baits in the field. We designed a device that promoted high rates of bait uptake by freshwater crocodiles (47% baits consumed), varanid lizards (19% baits consumed), and non‐target taxa (34% baits consumed) and our protocol could be honed to target specific predators at different times of day and different locations. We present the first conservation baiting protocol designed specifically for reptiles and our results demonstrate the feasibility of widespread and taxon‐specific deployment of aversion‐inducing baits to buffer the impacts of a toxic invasive species.

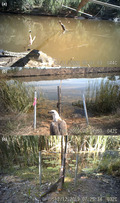

## 1 INTRODUCTION

Alien species can have devastating impacts on the ecosystems they invade, e.g., feral cats, *Felis catus*, across many countries (Loss & Marra, 2017), and eradicating invaders is often not feasible (Clout & Veitch, 2002; Pluess et al., 2012). An alternative management tactic to reduce the impacts of biological invasions is to modify the behavior of native species in ways that reduce their vulnerability (O'Donnell et al., 2010; Ward‐Fear et al., 2016). For example, low levels of exposure to invasive predators may facilitate the rapid adoption of antipredator responses, thereby enhancing survival of translocated fauna after release into the field (Edwards et al., 2021). One promising method of buffering the impact of an invasion involves the case where the invader is highly toxic and can be fatal if ingested by native predators. The most intensively studied example of this phenomenon involves the spread of cane toads (*Rhinella marina*) through tropical Australia (reviewed by Shine, 2018). Studies using vulnerable predators in captivity or radio‐tracked in the field have induced Conditioned Taste Aversion (CTA; Gustavson et al., 1974) by offering native predators a meal of toad flesh or a small live toad containing a sublethal dose of toxin (O'Donnell et al., 2010; Price‐Rees et al., 2013; Somaweera et al., 2011; Ward‐Fear et al., 2016). The resulting nausea teaches the predator to avoid future meals of toad flesh, thereby enhancing rates of survival in a toad‐infested habitat. For logistical reasons, the CTA training has been delivered to animals in captivity (that were subsequently released) in all but one of these studies; the sole exception being the study by Ward‐Fear et al. (2016), who presented free‐ranging (radio‐tracked) varanid lizards with small live toads.

Bringing predators into captivity or offering small toads individually to predators in the field cannot easily be upscaled to landscape‐level attempts at conservation via CTA. To deploy aversion‐inducing stimuli at a broad scale, management authorities can release large numbers of small live toads (see: www.canetoadcoalition.com). The success of this “teacher toad” method depends on multiple factors. The hunting strategy of the predator should include investigative handling of live anuran prey prior to consumption (as opposed to scavenging, or immediate seizure of prey). The predator should be small enough to ensure that the minute amount of toxin present in a metamorph toad elicits a negative experience (e.g., nausea). Lastly, to ensure encounters with metamorph toads, the predator should be diurnally active and forage terrestrially. In short, the method is suited to some but not all vulnerable predators (Ward‐Fear et al., 2017). Releasing live toads also runs the risk of public opposition (Shine et al., 2018) and potential problems with lack of control over deployment (if toads disperse rapidly from sites of release), ethical problems (causing fatal encounters between toads and predators), and collateral impact (if toads grow large enough to be fatally toxic).

To address these issues, ideally, we need to deploy non‐living baits to induce CTA, in a way that is time‐efficient and cost‐effective, while still providing a realistic “cue” that can be generalized by the predator to living adult cane toads. The method must work for timid predators at night as well as by day and be suitable for aquatic and semi‐aquatic species (not only terrestrial species, as in previous work). The methodology should be fine‐tuned to maximize the proportion of baits that are taken by vulnerable taxa rather than other predator and scavenger species that are unaffected by toad invasion and hence, do not benefit from CTA (e.g., most birds, rodents, invertebrates: Beckmann & Shine, 2009; Shine, 2010, 2018).

In this study, we address the issues of where, when, and how we can present aversion‐inducing baits to maximize opportunities to induce CTA in vulnerable predators. We targeted selected species by varying the location of baits (e.g., over water vs. over land), timing of deployment, and the force that a predator needs to exert to remove the bait from the apparatus.

Another critical issue for rates of bait uptake is the duration for which a bait remains attractive under a range of conditions (e.g., sun vs. shade). To explore these questions, we constructed bait delivery systems targeted at semi‐aquatic reptiles that are vulnerable to cane toad‐induced declines. We deployed bait stations at field sites in tropical Australia and conducted studies on captive reptiles to evaluate the forces they exerted while attempting to remove the bait from the apparatus.

In order to develop protocols that encourage predators to baits, we manipulated:

1. baiting location (water, water's edge, and bank) to target crocodiles and varanid lizards (and reduce offtake by non‐target species).
2. time of day to deploy baits (morning, afternoon, or both) based on patterns of bait offtake and balanced by rates of bait desiccation.
3. method of attaching baits to apparatus to further limit offtake by non‐target species, based on the pulling force required to remove it.

Based on our findings, we speculate on the predicted uptake of CTA in semi‐aquatic reptiles, through targeted conservation interventions in the wild, and identify knowledge gaps for future research.

## 2 METHODS

### 2.1 Study system

The Kimberley region, located in the monsoonal wet–dry tropics of northwestern Australia, experiences strong seasonality in rainfall patterns. Up to 95% of annual rainfall occurs in the “wet” season (November to April; long‐term average 798 mm: Bureau of Meteorology, 2020) rather than the “dry” season (May to October; long‐term average 35 mm: Bureau of Meteorology, 2020). The cane toad invasion frontline is advancing westwards through this region in the wet season, with toads becoming sedentary in the dry season (Shine et al., 2018).

**FIGURE 1:**
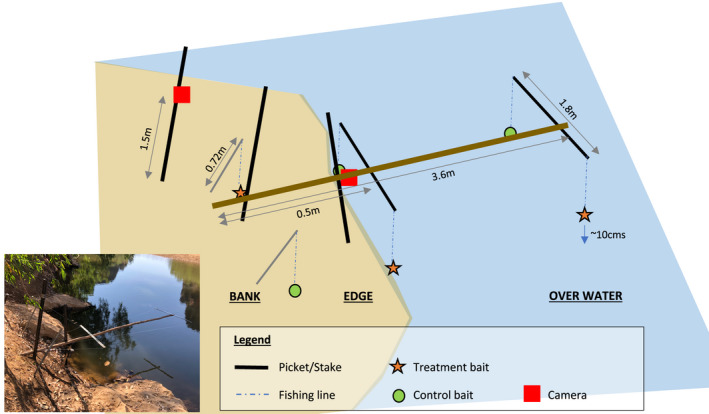
Diagram of baiting apparatus showing materials and measurements of apparatus components. Bait locations (over‐water, at the water's edge, and on the bank [on land]) and positioning of two remote cameras – one at the rear and one at the front of the apparatus are shown. Inset picture: image of baiting apparatus in the field. Photograph by M. Bruny

We targeted waterbodies near Kununurra in the eastern Kimberley (15°46′24″S, 128°44′21″E) and Windjana Gorge National Park in the central Kimberley (17°24′2″S, 124°56′4″E). At the time of the study, cane toads had invaded the former site but had not yet reached the latter. The waterbodies we used ranged from artificial gravel pits to waterbodies surrounded by woodland (Figure 1, Table 1). Trials were conducted in November 2019 (Kununurra) and September 2020 (Windjana Gorge). We chose five waterbodies in each area based on the presence of freshwater crocodiles (*Crocodylus johnstoni*) in spotlighting surveys (Fukuda et al., 2012). For safety reasons, sites that may have supported saltwater crocodiles (*C*. *porosus*) were excluded. Multiple varanid species are common across the region and have previously been detected at these locations during biodiversity surveys (G. Ward‐Fear, pers. obs.; DBCA, unpubl. data).

**TABLE 1:** Species recorded on camera visiting baiting stations by location

Population	Species group	Species	Scientific name
Windjana Gorge	Crocodiles	Freshwater crocodile	*Crocodylus johnstoni*
	Varanid lizards	Mertens' water monitor	*Varanus mertensi*
	Water birds	Brolga	*Grus rubicunda*
		Black‐fronted dotterel	*Elseyornis melanops*
	Birds	Bar‐shouldered dove	*Geopelia humeralis*
		Black bittern	*Ixobrychus flavicollis*
	Crustaceans	Giant freshwater prawn (Cherabin)	*Macrobrachium rosenbergii*
	Mammals	Agile wallaby	*Macropus agilis*
Kununurra	Crocodiles	Freshwater crocodile	*Crocodylus johnstoni*
	Varanid lizards	Mertens' water monitor	*Varanus mertensi*
		Mitchell's water monitor	*Varanus mitchelli*
	Raptors	Whistling Kite	*Haliastur sphenurus*
		White‐bellied sea eagle	*Haliaeetus leucogaster*
		Wedge‐tailed eagle	*Aquila audax*
	Water birds	Brolga	*Grus rubicunda*
		Great Egret	*Ardea alba*
		Comb‐crested Jacana	*Irediparra gallinacea*
	Mammals	Northern nail‐tail wallaby	*Onychogalea unguifera*
		Agile wallaby	*Macropus agilis*

### 2.2 Study species

Males of the Australian freshwater crocodile (*Crocodylus johnstoni*) grow to 3 m in snout‐to‐vent length (SVL) and up to 100 kg in mass; females grow to 2 m SVL and up to 40 kg (Britton, 2002). Freshwater crocodiles generally hunt small aquatic and semi‐aquatic prey including fish, amphibians, and aquatic reptiles in shallow water (Webb et al., 1982), but also occasionally take larger terrestrial prey (Somaweera et al., 2018; Webb et al., 1982). During the dry season, crocodiles become restricted to small, crowded pools (Tucker et al., 1996; Webb et al., 1982). Scarcity of food may induce some crocodiles to forage terrestrially, bringing them into more frequent contact with cane toads. Some populations of freshwater crocodiles have experienced significant declines, affecting specific size classes, due to lethal toxic ingestion of toads (Fukuda et al., 2016; Letnic et al., 2008; White, 2003), whereas other populations have been largely unaffected (Catling et al., 1999; Somaweera & Shine, 2012). The degree to which a population is impacted may depend upon biotic (e.g., food availability and population demography) and abiotic factors (e.g., climatic and hydrological variables and local ecology), which may explain why impacts have varied spatially and temporally (Fukuda et al., 2016; Somaweera et al., 2013). However, the ecological “crowding” phenomena and temporal overlap with toads provide an excellent opportunity to target large sections of the population with conservation interventions at critical times of vulnerability throughout the year.

Monitor lizards (family Varanidae) are among the largest terrestrial predators in tropical Australia and, like crocodiles, consume a wide diversity of prey (King & Green, 1999). Dietary composition varies seasonally and with lizard body size (King & Green, 1999; Ward‐Fear et al., 2020); anurans may be taken primarily in the wet season (Ward‐Fear et al., 2020). Varanids are diurnally active, and forage in both terrestrial and aquatic habitats (King & Green, 1999). Keen olfactory senses allow them to locate toads in their daytime shelters (G. Ward‐Fear, personal observation). Populations of large‐bodied species (yellow‐spotted monitor, *Varanus panoptes*; Mertens’ water monitor, *V*. *mertensi*) have declined by >90% following toad invasion (Brown et al., 2013; Doody et al., 2009; Shine, 2010), and Mitchell's water monitor (*Varanus mitchelli*) may be endangered due to toads (Laive et al., 2021).

Other predators and scavengers in these tropical sites include raptors such as brown kites, whistling kites, wedge‐tailed eagles, sea eagles, falcons, and goshawks, all of which are expected or have been confirmed to predate upon cane toads and scavenge the bodies of dead toads (Beckmann & Shine, 2009, 2011). Native meat ants (genus *Iridomyrmex*) also readily consume cane toads without ill effect (Ward‐Fear et al., 2010) and are abundant throughout tropical Australia.

### 2.3 Baiting apparatus design and deployment

To offer baits to predators, we constructed an apparatus consisting of a 3.6‐m central wooden pole (50 mm in thickness) extending over the waterbody and secured on the bank using metal stakes (see Figure 1). Cross bars secured to the central pole created paired attachment points for three baiting locations: hanging over the water (“over water”), at the “water edge” of the waterbody, and on land (“bank”). This set‐up constituted a single bait station. To increase attraction to the bait stations, we placed a control bait (~40 g chicken neck) and a treatment bait (~25 g rear half of adult cane toad carcass) at each baiting location. Because these trials aimed only to develop the methodology for bait deployment, we did not use toad baits that would elicit a CTA response in our naive populations (conservation interventions will take place in the future). As such, toads were rendered non‐lethal by the removal of internal organs and parotid glands (Chen et al., 2017) and we did not add additional nausea‐inducing agents such as lithium chloride (Price‐Rees et al., 2013). Baits were attached to the cross‐bars with 10 cm biodegradable cotton twine (safe for animals to consume; Ward‐Fear et al., 2016) tied around the bait and double knotted at the end for suspension by 19 mm bulldog clip. This clip grasped the outside of the knot and allowed easy release of the bait when pulled. The twine was attached to the apparatus by fishing line (23 kg tension, 0.8 mm diameter). Baits were suspended approximately 10 cm above the surface (land or water) to prevent contact with the substrate, and hence minimize consumption by ants or fish. As prey movement can trigger a feeding response in crocodiles (Grigg, 2015), we hung baits with slack line to allow them to move freely with the wind.

To identify animals foraging at the bait stations, we set up two remotely triggered wildlife camera traps (motion and infrared sensing; Model Ltl Acorn 6310Wmc) at each apparatus. One camera was set underneath the apparatus, facing out toward the “over water” baits. The other camera was secured to a vertical metal stake on the bank 1.5 m behind the apparatus to capture activity at the “bank” and “water edge” bait locations. As reptiles may show only a minimal thermal differential compared to water (Rovero et al., 2013; Welbourne et al., 2015), we used the highest setting for motion sensitivity. Cameras were set to take short videos (1 min) with every trigger, with no delay between triggers. We set up three baiting stations equally spaced around each waterbody, with absolute distances between adjacent bait stations depending on waterbody size (20 m to more than 50 m).

Each baiting trial lasted for 5 days, beginning at 17:00 h and concluding at 17:00 h on the 5th day. Bait types were randomly assigned to either the left‐hand side (LHS) or right‐hand sides (RHS) of the apparatus at each location, so that each location contained one bait of each type. Baits were checked each morning (AM: 07:00–09:00 h) and afternoon (PM: 16:00–18:00 h). We recorded which baits had been eaten from each location during the previous baiting session and replaced them. To freshen baits and encourage visitation, we replaced all baits at 17:00 h on Day 2, swapping their respective sides on the apparatus. In total, each field study location (Kununurra and Windjana Gorge) had 45 baits of each type on offer per baiting period (*n* = 5 waterbody locations, *n* = 3 bait stations per site, and *n* = 3 per bait station). Video data were collected for eight distinct time intervals: four full nights (5:00 p.m. to 8:00 a.m. the next day) and four full days (8:00 a.m. to 5:00 p.m.) for each bait station.

### 2.4 Processing and statistical analysis of field data

At each check, we classified each bait as either *eaten* or *uneaten*. Where possible, the consumer animal was identified using remote camera footage. When documenting predator habitat use and bait consumption, we analyzed data only from waterbodies where all three main types of predators (crocodiles, varanids, and raptors) were present (*n* = 5). To investigate crocodilian engagement with the apparatus, we included data from all waterbodies where crocodiles were present (with or without other predators; *n* = 10).

To test if different types of predators (crocodiles, varanids, and raptors) took baits at different locations (“over water,” “water edge,” and “bank”) and at different times of day (AM or PM baiting period), we ran a full factorial multinomial generalized linear mixed model (GLMM) with a logit function. We included “bait station ID” nested within “waterbody ID” as a random factor to account for pseudoreplication and repeated measures. These analyses were run in SPSS Version 26 (IBM, Armonk, New York, USA).

For all remote camera videos containing animals in frame, movements around the bait stations were classified into either visits (where animals showed no engagement with the apparatus) or interactions (where animals investigated, engaged with, or consumed a bait). Using one‐way analysis of variance tests (ANOVAs), we compared the mean bait *deployment*‐*to*‐*forage* time (as a measure of bait consumption latency) for each taxon. We then used Tukey–Kramer post hoc tests to compare differences among predator species. Chi‐squared tests were used to inform descriptive analyses including the percent of interactions vs. visits, and percent of nocturnal activity by taxa (nocturnal hours were defined as hours of darkness, 6:00 p.m. to 5:00 a.m.). These analyses were run in JMP 14.2.0 (SAS Institute).

### 2.5 Rates of bait desiccation

To quantify rates of toad bait desiccation in the field, we monitored baits that had been strung up in the sun (*n* = 5) and in the shade (*n* = 5). At intervals of 0, 3, 6, and 12 h, we retrieved baits briefly, weighed them to determine how much mass (water) they had lost, and used a metal probe to push on the thigh muscle at a constant and standardized pressure by hand. Once resistance was felt, we stopped and scored “bait hardness” (1 = soft to 4 = very hard). To analyze the data, we ran a full factorial ANOVA with the independent variables of treatment (sun vs. shade), time since bait deployment (0, 3, 6, and 12 h), and the interaction between the two. The dependent variables were cumulative mass decrease as a percentage of initial bait mass and the index of bait softness. We included bait ID as a random factor to account for repeated measures.

### 2.6 “*Pull force*” exerted by captive reptiles on bait mechanisms

To gauge the force required by different taxa to dislodge a bait from the apparatus (and thus hone the methodology for bait attachment), we measured the pull force of varanid lizards (*Varanus giganteus*, *V*. *spenceri*, and *V*. *varius*) and freshwater crocodiles during routine feeding sessions with captive animals at two wildlife parks (The Australian Reptile Park and Hartley's Crocodile Adventures). A digital pull–push force gauge (BYQTEC model FM‐204) was attached to the base of a hand‐held metal feeding pole using a carabiner. Following normal husbandry protocols, animal keepers attached 15 cm of biodegradable cotton twine to the short axis of 100 g piece of raw beef (the animals’ regular food) using a double knot. The other end was tied to the sensing screw hook on the force gauge. The peak reading for the pull force exerted was recorded as each animal attempted to take the bait. If the twine did not break, the keepers cut it so that the animal could consume the bait. Any other pertinent behaviors were noted, and body lengths (SVL, mm) for individual animals were obtained from husbandry records. Neither the hook nor gauge came into contact with the animal at any time. We used linear regression analyses to determine the relationships between body size (SVL) and peak pull force (Newtons) for both varanids and crocodiles.

## 3 RESULTS

A total of 15 vertebrate species visited our bait stations (see Table 1 for a list). The most frequent consumers of baits were crocodiles (47%), followed by raptors (35%) and varanid lizards (19%). In total, 49.8% of our baits were consumed (55.6% of 693 toad bait opportunities and 43.9% of 693 chicken bait opportunities).

### 3.1 Animal activity

#### 3.1.1 Crocodiles

**FIGURE 2:**
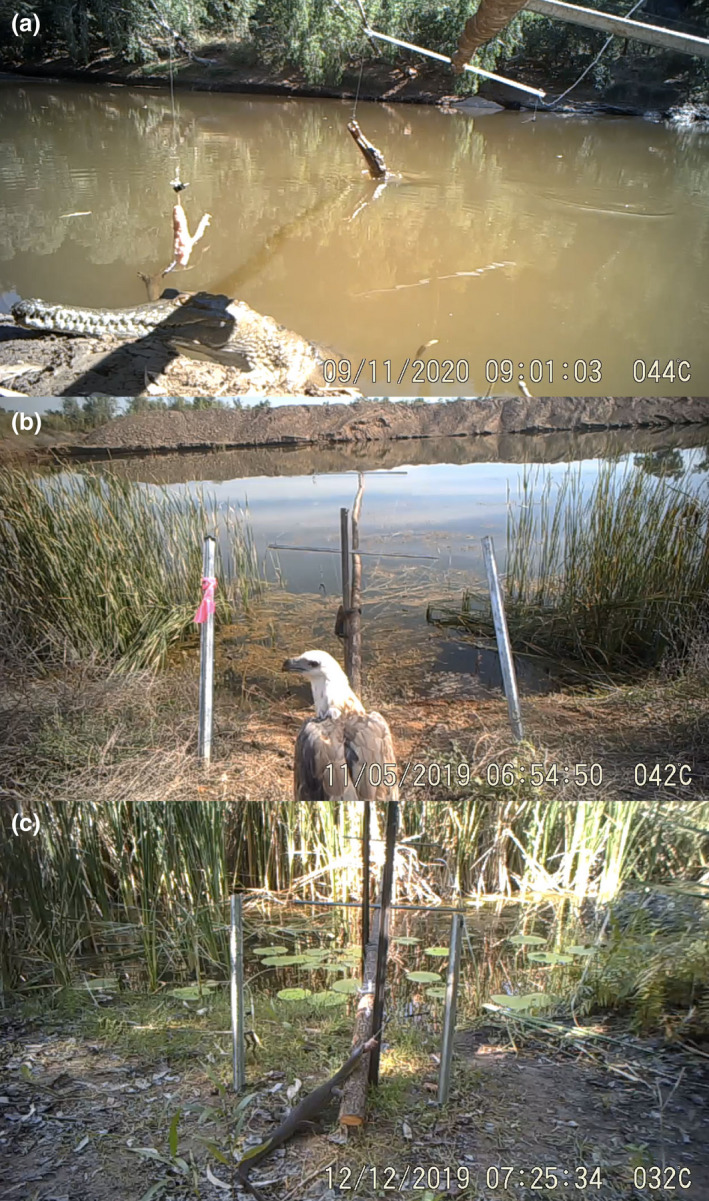
Interactions with the apparatus by predators during field trials: (a) two freshwater crocodiles (*Crocodylus johnstoni*) visiting the same bait station; (b) a raptor (white‐bellied sea eagle, *Haliaeetus leucogaster*); and (c) a varanid lizard (Mertens’ water monitor, *Varanus mertensi*). Photographs taken using Ltl Acorn 6310Wmc remote camera traps at the forward (a) location, and behind positions (b and c)

A total of 1636 videos of freshwater crocodiles close to baiting stations were captured on camera (each 1 min long; see Figure 2a), and crocodiles interacted with the bait apparatus on 63% of occasions. Behaviors included both investigation (e.g., sniffing and poking) and consumption or attempted consumption of baits. The remaining occasions (37%) were classified as visits and included instances of basking, resting, or using the area to move between water and land.

#### 3.1.2 Varanid lizards

Three species of varanids were recorded on videos: yellow‐spotted monitor, *Varanus panoptes* (10 occasions); Mertens’ water monitor, *V*. *mertensi* (47 occasions); and Mitchell's water monitor, *V*. *mitchelli* (12 occasions) (Figure 2b). Varanids interacted with baits 55% of the time. The remaining occasions (45%) were classed as visits and included instances of swimming, climbing on the apparatus to bask, and walking or resting within close proximity.

#### 3.1.3 Raptors

Three species of raptors were also recorded: white bellied sea eagle, *Haliaeetus leucogaster* (9 occasions); wedge‐tailed eagle, *Aquila audax* (22 occasions); and whistling kite, *Haliastur sphenurus* (8 occasions) (Figure 2c). Raptors interacted with baits 62% of the time. The remaining occasions (38%) included instances of perching on the apparatus, resting, and preening within close proximity.

#### 3.1.4 Meat ants

We found meat ants (*Iridomyrmex reburrus*) on 24 uneaten baits (12 toad, 12 chicken). We opportunistically witnessed (on camera) meat ants on fresh baits that were subsequently eaten, suggesting that meat ants consumed even more baits despite design features intended to deter them. From our direct observations, meat ants foraged baits both day and night and located 50% of baits within 3 h of deployment. Ants preferentially exploited baits located on the bank (58%) followed by the water's edge (33%); they also utilized the apparatus to access baits positioned over the water (8%). On one occasion, so many meat ants swarmed the apparatus that we had to remove all baits for the day, until the ants had retreated. We cannot infer how strongly the presence of meat ants deterred other animals from consuming baits, although our cameras captured crocodiles eating baits that were being consumed by meat ants simultaneously. Because bait consumption by meat ants and other animals may not be mutually exclusive, our analyses focused only on comparisons of bait consumption by competing taxa of vertebrate predators (i.e., raptors, varanids, and crocodiles).

### 3.2 Location of bait consumption by different types of predators

**FIGURE 3:**
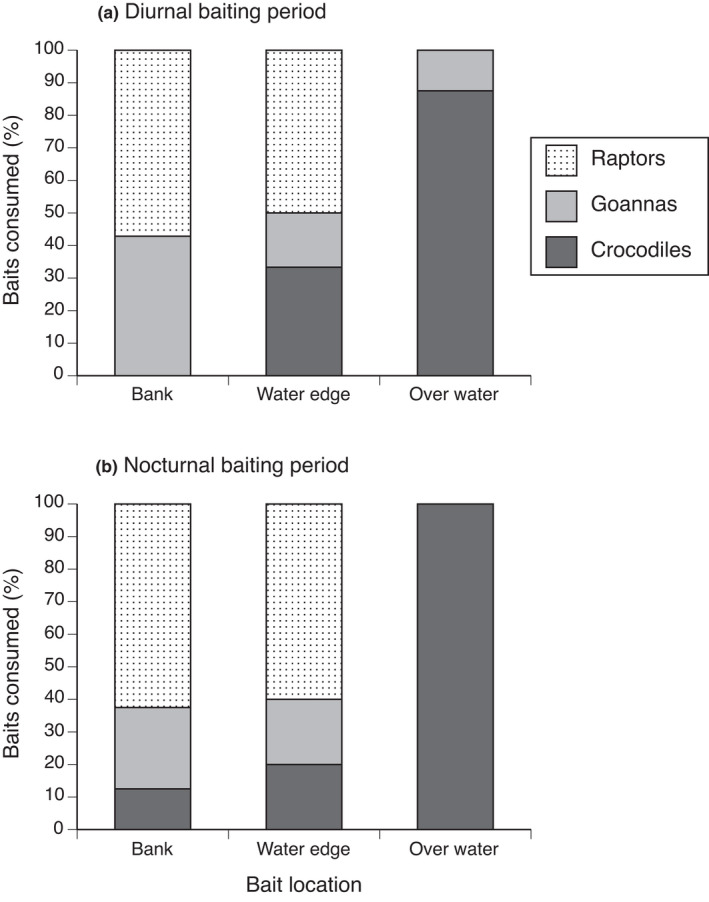
Proportion of total consumed baits at each location by different predators (crocodiles, varanid lizards [goannas], and raptors) during the (a) diurnal baiting period (6:00 a.m. to 5:00 p.m.) and (b) the nocturnal baiting period (5:00 p.m. to 6:00 a.m.)

Different predator groups consumed baits at different locations (*F*_4,47_ = 3.62, *p* = .013). Goannas and raptors predominantly foraged on the bank and at the edge of the waterbody with few baits taken from over the water (Figure 3). The average bait *deployment*‐*to*‐*forage* time differed among predators, with varanids locating baits the soonest after deployment (mean time of 275 min), followed by crocodiles (mean time of 343 min) and raptors (mean time of 411 min) (*F*_2,132_ = 3.26, *p* = .042).

### 3.3 Timing of bait consumption by different types of predators

The baiting period (day or night) did not significantly affect which predators consumed baits (*F*_2,47_ = 0.82, *p* = .448) or where they consumed baits (*F*_2,47_ = 0.19, *p* = .824). Predators visited stations both diurnally and nocturnally, with differential habitat use by time of day. For example, crocodiles were more likely to visit baits on the bank at night (Figure 3). Overall, crocodiles were equally active around bait stations diurnally (48% of their total activity) and nocturnally (52% of total activity), whereas varanids and raptors were mostly diurnal (91% of total activity; X^2^ = 93.73, df =1, *n* = 901, *p* < .0001) with minimal nocturnal activity (9% total raptor + varanid activity; 91% of which were raptors, the remaining 9% were varanid lizards).

### 3.4 Rates of bait desiccation

The interaction between treatment (sun vs. shade) and duration of bait deployment was significant for both percent mass loss over time (*F*_7,39_ = 8.4, *p* < .0003) and bait hardness (*F*_2,47_ = 28.6, *p* < .0001). All baits desiccated significantly after 12 h, but baits in the sun did so much faster than those in the shade. After 3 h, sun‐exposed baits were physically harder and had lost an average of 31% mass compared to the softer baits in the shade with 19% mass loss. After 6 h, sun‐exposed baits remained harder and had lost 48% cumulative mass, whereas shade baits had lost 36% on average. By 12 h, sun‐exposed and shaded baits were no longer distinguishable in terms of hardness, yet sun‐exposed baits had lost an average of 60% of their initial mass, compared to 52% for shaded baits.

In the field baiting trials, the majority of toad baits that were eaten were consumed quickly post‐deployment (i.e., within 10 min to 3 h) based on video footage and opportunistic checks of waterbodies post‐baiting. The only taxa to consume toad baits over longer periods were meat ants. Conversely, all taxa readily consumed the control chicken baits a full day after deployment.

### 3.5 “*Pull force*” exerted by captive reptiles on bait delivery mechanisms

Larger animals exerted stronger pull force, irrespective of species (*F*_1,58_ = 51.8, *p* < .0001). The highest peak reading for a varanid lizard was 65.8 Newtons, delivered by the largest animal tested (610 mm SVL), and the lowest was 14.9 Newtons by the smallest animal tested (400 mm SVL). For crocodiles, the peak reading was 149.9 Newtons for an animal of SVL 1250 mm and the lowest was 21.33 Newtons by a slightly larger animal of 1300 mm SVL.

## 4 DISCUSSION

Our novel method of delivering food items to free‐ranging tropical predators resulted in frequent bait uptake and most of that uptake was by target species (overall, crocodiles, and varanid lizards took 65% of baits). Our apparatus allowed feeding by multiple predators at once (Figure 2a) and repeat ingestion of CTA baits (which may speed up and reinforce learning experiences). Unsurprisingly, in this highly biodiverse area, other native species (notably, raptors, and ants) also consumed baits. Importantly, however, judicious placements of baits (e.g., over water and at night) substantially reduced rates of offtake by these non‐target species. Bait stations proved to be robust, rarely requiring repair or maintenance, and associated costs were low.

Another successful part of this methodology was the use of remotely triggered video cameras to film the approach of predators to bait stations. Previous studies have reported that such video systems can perform poorly for ectothermic animals because low thermal differentials between the animal and their background fail to trigger the cameras (Vaca‐Castano et al., 2019). In our study, numerous instances of bait offtake by both crocodiles and varanid lizards as well as by endotherms (birds) were captured.

Our study also identifies the duration of time for which a bait remains attractive to predators after initial deployment. Baits appear to remain palatable for up to 6 h, particularly if they are placed out of direct sunlight. Although some types of animal‐based defensive toxins breakdown rapidly post mortem (Phillips & Shine, 2007), the bufadienolide toxins of cane toads are highly thermostable (Crossland et al., 2011), as are the additional nausea‐inducing compounds often added to taste‐aversion baits to elicit stronger responses by native predators (Price‐Rees et al., 2011). Thus, the CTA‐inducing ability of baits should persist for as long as baits remain palatable and identifiable (via olfactory and visual cues) to predators. However, given the reasonably fast rates of bait desiccation, ideally baits would be deployed in the afternoon to ensure cue recognition. We saw that most baits were eaten within a few hours of deployment, thus if animals are present in the vicinity, they will likely find the baits within their most palatable timeframe.

Minor modifications to the bait station design might further facilitate targeting and increase the duration of bait attractivity (thus requiring less frequent replenishment). Shading of the bait could achieve the latter aim, whereas adjusting the strength of bulldog clips holding baits (and thus, the force required to remove a bait from the apparatus) offers a way to restrict bait offtake to larger predators (e.g., to crocodiles rather than smaller varanid lizards). That size selectivity could be useful not only to focus CTA experiences on the predators most vulnerable to cane toad impacts but also to ensure that small‐bodied predators, potentially at risk of fatal poisoning from even a small dose of toad toxin, are unable to obtain access to the baits. We have no data on “pull strength” of raptors, but it is likely to be less than that exerted by the larger reptiles; and if so, adjusting the force needed to remove a bait might offer a robust way to reduce offtake by raptors also.

Other modifications to the baiting system could also reduce non‐target offtake and bait spoilage. For example, although scavenging ants did not appear to dissuade crocodiles from taking baits, it would be straightforward to eliminate ant presence by placing the support stakes directly into shallow water rather than on the bank. Alternatively, ants could be discouraged by applying ant‐specific pesticides (e.g., Coopex) at the base of support stakes; or the ants could be encouraged to feed elsewhere by deploying alternative food on the edges of the waterbody (see Ward‐Fear et al., 2010). Although they occur in the study sites (ALA, 2021), we did not record dingos or feral pigs at our baits. These non‐target species likely could be dissuaded by fixing bait poles into the water.

How could this be “rolled out” as an on‐ground conservation strategy? Deploying baits by hand is less time‐ and cost‐effective than deploying baits aerially or via bait dispensers, methods often used in land management. However, unlike conservation programs which aim to kill feral animals, these baits aim to train native reptilian predators using taste aversion; thus, the bait must be realistic (i.e., more fresh). Studies on taste aversion to cane toads have shown that native predators learn rapidly (O'Donnell et al., 2010; Price‐Rees et al., 2013; Somaweera et al., 2011; Ward‐Fear et al., 2016). If timed to coincide with the arrival of the cane toad invasion, perhaps only one multiday training session would be required. Importantly, when nausea‐inducing chemicals are added to enhance learning (e.g., lithium chloride and thiabendazole), dosed baits must be targeted to animals of a certain body size. Thus, broad‐scale deployment of baits via helicopter throughout the environment may not be ethical or practical. Instead, crocodiles in particular should be targeted in the water for the best outcomes. Interventions could be run for a week at a time, with one round of baits delivered by a land manager each afternoon, and thus multiples sites could be run concurrently. There would be no need to set up the whole apparatus; stakes in the water would be sufficient. Managers would first identify areas of high biodiversity in the landscape to ensure a high cost‐to‐benefit ratio, as in most conservation programs where populations of concern are targeted by land management agencies.

Future research could also fine‐tune the seasonal delivery of baits to increase rates of uptake. The tropical dry season may be the time of greatest vulnerability of crocodiles to cane toads (Letnic et al., 2008), suggesting that deployment early in the dry season might be a suitable time for aversion training (i.e., minimizing the delay between training and encounter with live cane toads). For varanid lizards, in contrast, impacts of toads appear to be greatest during the wet season, the time when these lizards are most active (Ward‐Fear et al., 2016). Deployment of CTA stimuli late in the dry season, coincident with the first monsoonal storms, may reduce vulnerability of these lizards.

Our study was a first step in proof of concept to conservation interventions planned in the future; that is, our experimental design aimed at achieving high rates of bait uptake, rather than inducing conditioned taste aversion (CTA) in predators. For that reason, the baits we used were not sufficiently nausea inducing to result in CTA, and we have only presented the methodological considerations for a baiting protocol here. In terms of evaluating the value of our method for conservation intervention, however, it is worth considering whether the rates of bait uptake achieved in our study were high enough to induce CTA in a sufficiently high proportion of predators to buffer the impacts of toad invasion. To answer that question, we need data on four issues:

1. What proportion of predators in the populations we studied took our baits? Because animals were not individually marked, we cannot quantify exact numbers. Nonetheless, we documented animals (especially crocodiles) of a range of body sizes take baits from each location; and often, baits were taken from widely separated areas of each waterbody at the same time. Thus, multiple individuals were certainly involved.
2. What proportion of predators that took baits would have developed CTA if the baits had been nausea inducing? Published studies show that CTA can be induced readily, such that most or all of the animals used in our experimental trials exhibited significant aversion to cane toads after a single exposure to a nausea‐inducing bait (e.g., crocodiles—Somaweera et al., 2011; varanid lizards—Ward‐Fear et al., 2016; quolls—O'Donnell et al., 2010).
3. What proportional increment in survival (due to CTA) results in a significant enhancement of a population's ability to persist following invasion of toxic toads? Population viability analyses (PVAs) show that the answer to this question depends upon the species’ life‐history traits (such as age at sexual maturity, reproductive output, and longevity), but that even a minor increase in rates of adult survival can massively increase the population viability of a long‐lived taxon. For example, a 10% increase in adult survival can change a population trajectory from strongly negative to strongly positive (Desbiez et al., 2020). For a shorter‐lived species (as may be true of varanid lizards), population trends are less sensitive to adult survivorship; but even for such taxa, any significant increment in adult survival is likely to substantially enhance population viability, especially if reproductive output and juvenile recruitment is high.
4. What level of numerical buffering of toad impact, at a population level, would constitute “success” for a conservation intervention? There is no simple answer to that question, but reports of toad‐induced declines of >90% in populations of crocodiles (Letnic et al., 2008) and varanid lizards (Brown et al., 2013) suggest that even a modest increase in survivorship could facilitate population persistence, especially where conservation interventions could preserve the minimum viable population size for these predators. Thus, for example, if 50% of the predators learned to avoid toads, and hence survived rather than being fatally poisoned, the magnitude of population decline would be reduced fivefold (i.e., 50% rather than 10% survival).

These results suggest that the rates of bait uptake in our study likely were high enough to significantly buffer predator populations against toad invasion, particularly if we can hone methodology to increase uptake by target predators in the future, but further work is needed to empirically test that inference. Such studies are already underway, as the toad invasion moves through some of the last biodiversity hotspots in tropical Australia. Taste aversion currently represents the most promising tool to mitigate the impact of cane toads on native fauna, and our study helps inform best practice for rolling it out on the landscape level.

We initially designed this system to deliver CTA‐inducing baits to vulnerable predators, which (in addition to Australia) has application in other ecosystems experiencing invasions by toxic species (such as Asian black‐spined toads in Madagascar; Marshall et al., 2018). However, a standardized method of presenting edible prey to free‐ranging predators may be of value in many contexts. For example, our bait stations may facilitate research on the attributes of potential prey items that elicit feeding responses by a particular type of predator, or by subgroups (sexes and sizes) within a predator population. Similarly, investigators may wish to understand how the spatial and temporal availability of prey influence uptake rates by different types of predators. In the present study, we examined this question in the context of CTA training; but the same issue is relevant to topics such as resource partitioning within predator guilds (Luiselli, 2006). Yet, another potential use of the system is to explore how rates and locations of foraging by different types of predators are modified by factors such as local landscapes, seasons, and the arrival of invasive species. In short, knowing what kinds of predators attack a standardized bait, and the spatial and temporal predictors of those attack rates, can provide insights into a wide variety of fundamental issues in vertebrate ecology.

We designed our system not only to keep baits away from the substrate (ground or water) partly to keep them away from scavenging ants but also to produce a prey stimulus that was both highly visible and mobile and therefore, likely to attract the attention of a predator. Other studies that have offered prey items to free‐ranging predators typically place bait on the ground (Beckmann & Shine, 2011; Taylor et al., 2005). A system of suspended baits might offer additional opportunities to attract predators, for example, if baits also act as “lures” from a distance, although it would need to take into account the specific ecology of the target organisms. The same issues apply if the baits are used for feral animal control rather than research or conservation. Depending on the predator's habitat use and foraging behavior, a suspended bait may have advantages over one that is deployed on or under the ground.

In many places around the world, conservation baiting programs deliver poisonous baits to invasive mammalian species (e.g., rodents, mustelids, rabbits, cats, foxes, pigs, dogs, and possums: Allsop et al., 2017). Here, we present the first baiting methodology designed specifically for reptiles, including aquatic species, but excitingly, one which delivers “training” baits to increase rather than decrease the survival of target species. Our study confirms the feasibility of delivering a taste‐aversion stimulus in the field to free‐ranging crocodiles and varanid lizards. With some simple modifications of the timing and placement of baits, selectivity of uptake by specific types of predators can be enhanced. With refinements, our design may prove appropriate for a wide variety of uses related to research, management, and the conservation of threatened species.

## AUTHOR CONTRIBUTIONS

**Abhilasha Aiyer:** Conceptualization (equal); Data curation (equal); Formal analysis (equal); Investigation (equal); Methodology (equal); Writing – original draft (equal); Writing – review & editing (equal). **Bunuba Rangers:** Data curation (equal); Investigation (equal); Methodology (equal); Resources (equal). **Tina Bell:** Conceptualization (equal); Funding acquisition (supporting); Methodology (equal); Project administration (equal); Resources (equal); Supervision (equal); Writing – review & editing (equal). **Richard Shine:** Conceptualization (equal); Formal analysis (equal); Funding acquisition (equal); Investigation (equal); Methodology (equal); Resources (equal); Supervision (equal); Writing – original draft (equal); Writing – review & editing (equal). **Ruchira Somaweera:** Conceptualization (equal); Investigation (equal); Methodology (equal); Supervision (equal); Writing – original draft (equal); Writing – review & editing (equal). **Miles Bruny:** Data curation (equal); Investigation (equal); Methodology (equal); Project administration (equal); Resources (supporting). **Georgia Ward‐Fear:** Conceptualization (equal); Data curation (equal); Formal analysis (equal); Funding acquisition (equal); Investigation (equal); Methodology (equal); Project administration (equal); Resources (equal); Supervision (equal); Writing – original draft (equal); Writing – review & editing (equal).

## CONFLICT OF INTEREST

The authors declare no conflicts of interest.

## Data Availability

The data that support the findings of this study are available from the Dryad Digital Repository at https://doi.org/10.5061/dryad.cfxpnvx7j.
